# Impact of super resolution deep learning reconstruction with a 1024 matrix on CAD-RADS classification of intermediate coronary stenosis: A bicentric retrospective study

**DOI:** 10.1016/j.ejro.2026.100821

**Published:** 2026-10-26

**Authors:** Jean Philippe Dillenseger, Fuminari Tatsugami, Aissam Labani, Wataru Fukumoto, Soraya El Ghannudi, Akira Taniguchi, Karim Haioun, Catherine Roy, Kazuo Awai, Mickaël Ohana

**Affiliations:** a Pole d’imagerie médicale, Hôpitaux universitaire de Strasbourg, Strasbourg, France; b ICube – UMR 7357, CNRS, Université de Strasbourg, Strasbourg, France; c Faculté de médecine, maïeutique et des sciences de la santé, Université de Strasbourg, Strasbourg, France; d Department of Diagnostic Radiology, Graduate School of Biomedical Health Science, Hiroshima University, Japan; e Canon Inc., Shimomaruko, Tokyo, 146-8501, Japan

**Keywords:** Coronary Artery Disease, X-Ray Computed tomography, Deep Learning, Image reconstruction

## Abstract

**Introduction:**

Moderate coronary stenosis (CAD-RADS 3) is a diagnostic gray zone in coronary CT angiography (CCTA). We evaluated whether super-resolution deep learning reconstruction (SR-DLR), particularly with a 1024 matrix, modifies CAD-RADS classification and inter-reader agreement. Materials and methods: Thirty-nine CCTA examinations from two centers, each containing at least one 50–69% stenosis identified on the routine normal-resolution deep learning reconstruction (NR-DLR), were reconstructed with NR-DLR and SR-DLR (512 and 1024 matrices). Five experienced readers blindly assigned CAD-RADS categories. Agreement was assessed with Gwet’s AC2 and paired reclassification with exact McNemar tests. Subgroup analyses (plaque composition, initial image quality) were exploratory.

**Results:**

Forty-six lesions were analyzed (19 calcified, 16 mixed, 10 non-calcified, one in-stent). Agreement remained excellent (AC2 0.89 [0.86–0.92], 0.87 [0.83–0.90] and 0.84 [0.81–0.88] for NR-DLR, SR-DLR-512 and SR-DLR-1024). Consensus CAD-RADS 2 rose from 6.5% to 21.7% with SR-DLR-1024 (p = 0.016; 10 lesions downgraded, 2 upgraded), predominantly in lesions with non-calcified components. Per-reader CAD-RADS 2 rates ranged from 13% to 30%, increasing significantly for two of five readers (p = 0.004).

**Conclusion:**

SR-DLR-1024 modified CAD-RADS classification in about one fifth of intermediate lesions while preserving excellent agreement. Without an invasive reference standard, these reclassifications reflect a change in reader interpretation rather than a proven gain in accuracy.

## Highlights

• SR-DLR with a 1024 matrix influences CAD-RADS classification in CCTA.
• CAD-RADS 2 consensus rate rises with SR-DLR-1024 (22% vs 7%; p = 0.016).
• Inter-reader agreement remains excellent across all reconstructions.
• Downgrading is numerically more frequent in lesions with a non-calcified component.
• SR-DLR is a software-based approach requiring no dedicated hardware.

## 1 Introduction

Coronary CT angiography (CCTA) has become the first-line non-invasive imaging modality for evaluating suspected coronary artery disease because of its excellent negative predictive value and its central role in current diagnostic pathways [1]. Beyond the detection of obstructive disease, CCTA is also the only non-invasive modality able to identify and characterise non-obstructive coronary atherosclerosis, the extent and composition of which carry independent prognostic value, as demonstrated by multicentre studies such as the CAPIRE study and the PARADIGM registry [2], [3]. Standardized reporting using the Coronary Artery Disease-Reporting and Data System (CAD-RADS) has improved communication by linking stenosis severity to management recommendations [1]. However, visual assessment remains challenging for intermediate lesions classified as CAD-RADS 3 (50–69% luminal diameter reduction), one of the main diagnostic gray zones in CCTA. Small differences in visual estimation around this threshold may alter downstream management, including functional testing, invasive coronary angiography, or medical therapy.

Assessment of intermediate stenoses is limited not only by lesion severity but also by the spatial resolution of the imaging system, image noise, motion artefacts, and partial-volume effects. Blooming from coronary calcifications may overestimate stenosis severity, while even non-calcified plaques become difficult to evaluate when the residual lumen approaches the spatial resolution limits of conventional CCTA [4], [5]. Technologies capable of improving effective spatial resolution have therefore attracted considerable interest.

Dedicated ultra-high-resolution CT (UHR-CT) and, more recently, photon-counting CT (PCCT) have improved visualization of the coronary lumen and plaque morphology by increasing spatial resolution and reducing partial-volume effects [4], [5], [6], [7], [8]. These systems show better agreement with invasive coronary angiography and more frequent downgrading of intermediate stenoses, particularly in calcified vessels [5], [6], [7], [8]. However, their clinical implementation remains limited by cost and availability.

An alternative approach is to enhance image resolution during reconstruction rather than acquisition. Super-Resolution Deep Learning Reconstruction (SR-DLR; Precise IQ Engine, Canon Inc., Otawara, Tochigi, Japan) is a commercially available algorithm trained with paired standard- and ultra-high-resolution datasets to generate images approaching dedicated UHR-CT while preserving conventional CCTA workflows [9], [10], [11]. Phantom and early clinical studies have demonstrated improvements in spatial resolution, edge sharpness and overall image quality compared with conventional deep learning reconstruction [9], [12], [13], [14], [15], [16], [17], [18], [19], [20], [21], [22]. More recently, Zou et al. reported that SR-DLR modified CAD-RADS classification in approximately 20% of patients and improved diagnostic performance compared with hybrid iterative reconstruction, using invasive coronary angiography as the reference standard [23], suggesting that the reconstruction strategy itself may influence visual stenosis assessment.

Nevertheless, several questions remain. The respective contributions of matrix size and super-resolution reconstruction to CAD-RADS classification are unclear, the impact of SR-DLR on inter-reader reproducibility has received little attention, and its behavior in diagnostically challenging intermediate CAD-RADS 3 lesions has not been specifically investigated.

The aim of this study was to evaluate the influence of SR-DLR on CAD-RADS classification of lesions initially identified as potential CAD-RADS 3 on conventional reconstruction. Conventional deep learning reconstruction was compared with SR-DLR reconstructed using 512- and 1024-matrix sizes to assess their impact on reader classification, inter-reader agreement, and lesion reclassification according to plaque composition. Rather than assessing diagnostic accuracy against an invasive reference standard, this study examined how advanced reconstruction techniques influence reader interpretation within one of the most clinically relevant gray zones of coronary CT angiography.

## 2 Methods

Institutional Review Board approval was obtained for the present study. Given its retrospective nature, signed patient consent was waived.

### 2.1 Study population

Between November 2022 and January 2023, consecutive patients who underwent a clinically indicated CCTA in the two participating university hospitals were retrospectively screened (Centre 1: n = 29 patients / 33 lesions; Centre 2: n = 10 patients / 13 lesions). Patients were eligible when the original clinical report, established on the normal-resolution deep learning reconstruction (NR-DLR, AiCE) used in routine practice in both centres, described at least one intermediate 50–69% luminal stenosis (i.e. a potential CAD-RADS 3 lesion), and when raw projection data were still available to allow additional reconstructions. No additional exclusion criteria were applied in order to preserve the consecutive nature of the cohort and reflect routine clinical practice.

It should therefore be emphasized that the study population was intentionally enriched with lesions initially considered intermediate on conventional reconstruction. Consequently, the objective of the present work was not to estimate the prevalence of CAD-RADS reclassification in an unselected CCTA population but rather to evaluate the influence of alternative reconstruction strategies on lesion classification within this predefined clinically relevant subgroup.

For each examination, the following variables were recorded:

- patient age, sex and body weight;

- dose-length product (DLP);

- calcium score;

- image quality, rated as "good", "moderate" or "poor" in the initial local reading,

- for each moderate 50–69% stenosis: the vessel involved (LM, LAD, D1, Cx or RCA) and the type of stenosis (calcified, non-calcified, mixed, in-stent).

### 2.2 Coronary CT Angiography (CCTA)

All examinations were acquired on a 4th generation 320-row scanner, following the acquisition parameters summarized in Table 1. All included CCTA were reconstructed at the best phase (with the least cardiac motion) for the vessel with the intermediate stenosis (best phases were selected by the same experienced reader), using 3 different reconstruction algorithms Table 1:

**Table 1:** Acquisition and reconstruction parameters.

**Parameter**	**Value**
Manufacturer	Canon Inc.
Model	Aquilion ONE
Focal spot dimensions (mm2)	1.6 × 1.5
kVp	100 kV
mA	Automatic tube current modulation (SUREExposure)
Rotation time (s)	0.275
Detector characteristics	PUREViSION, 320 rows, row thickness 0.5 mm
Collimation in z direction (mm)	320 × 0.5 mm = 160 mm
Acquisition mode	Volume
Reconstruction algorithm	NR-DLR (AiCE) / SR-DLR-512 (PIQE 512) / SR-DLR-1024 (PIQE 1024)
FOV diameter; matrix; pixel	256 mm; 512 × 512; 0.5 × 0.5 mm2 / 256 mm; 512 × 512; 0.5 × 0.5 mm2 / 256 mm; 1024 × 1024; 0.25 × 0.25 mm2
Reconstructed slice thickness/interval (mm)	0.5 / 0.25 (all three reconstructions)

AiCE = Advanced intelligent Clear-IQ Engine; DLR = deep learning reconstruction; FOV = field of view; kVp = peak kilovoltage; mA = milliampere; NR-DLR = normal-resolution deep learning reconstruction; PIQE = Precise IQ Engine; SR-DLR = super-resolution deep learning reconstruction.

- Normal Resolution Deep Learning Reconstruction in 512 matrix size (NR-DLR),

- Super Resolution Deep Learning Reconstruction in 512 matrix size (SR-DLR 512),

- and Super Resolution Deep Learning Reconstruction in 1024 matrix size (SR-DLR 1024).

To minimize bias, the same DLR strength level (standard) was used for all reconstructions. NR-DLR (AiCE, standard strength, 512 matrix, 0.5 mm slice thickness) corresponds strictly to the reconstruction routinely used in both centres and on which the initial 50–69% stenosis was reported; it therefore constitutes both the "original" clinical reconstruction and the reference reconstruction for all subsequent comparisons. NR-DLR images are displayed in the upper row of Fig. 1, Fig. 2, Fig. 3, Fig. 4, Fig. 5, Fig. 6. All comparisons were based on the blinded re-reading of the NR-DLR datasets performed within the study, and not on the original clinical report, so that the three reconstructions were graded under identical conditions; this design also limits the contribution of regression towards the mean, inherent to any selection based on a threshold category, to the observed reclassification.

**Fig. 1:**
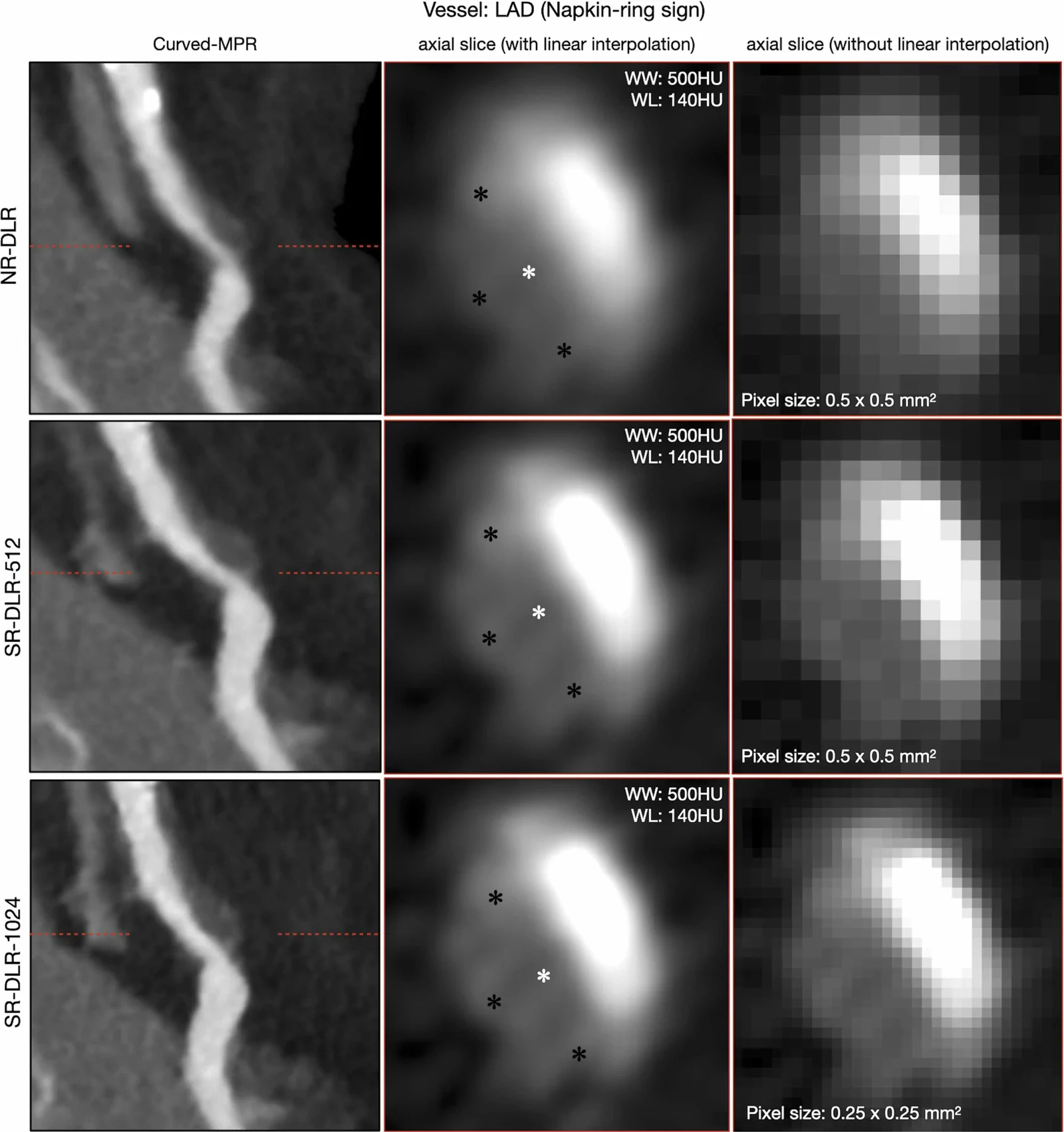
NR-DLR versus SR-DLR reconstructions in 512 and 1024 matrix. Differences in native pixel size are evident in interpolated trans-axial slices. The contrast between the enhancing ring surrounding a low-attenuation plaque area (black stars), and the dark region of low attenuation core (white star) appears more pronounced with SR-DLR 1024, findings that are suggestive of napkin-ring sign, one of the high-risk plaque features defined by CAD-RADS 2.0. Quantitative image quality assessment was not performed in this study.

**Fig. 2:**
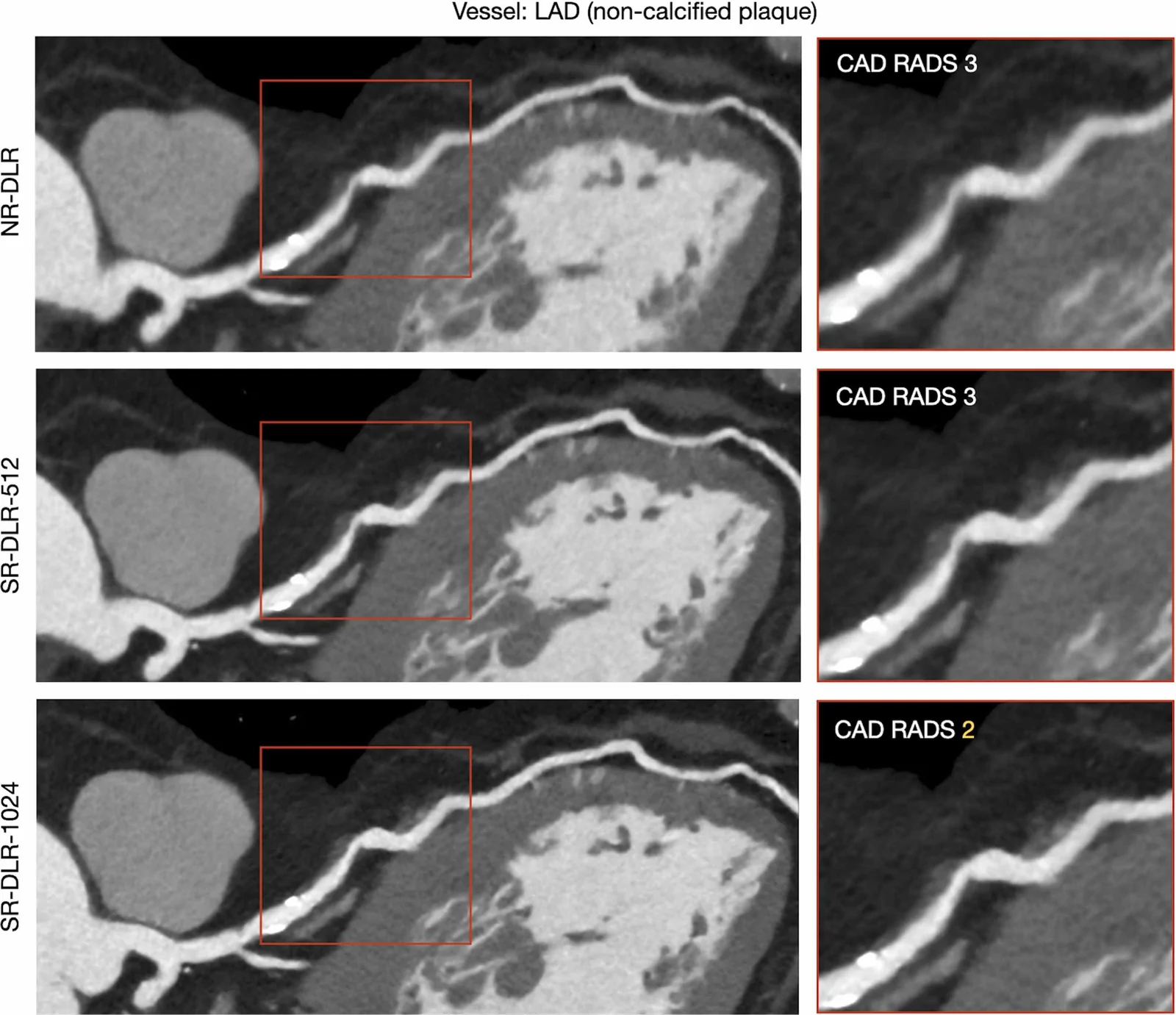
NR-DLR versus SR-DLR reconstructions in 512 and 1024 matrix. A downgrading of CAD-RADS classification (from 3 to 2) was established between SR-DLR-512 and SR-DLR-1024.

**Fig. 3:**
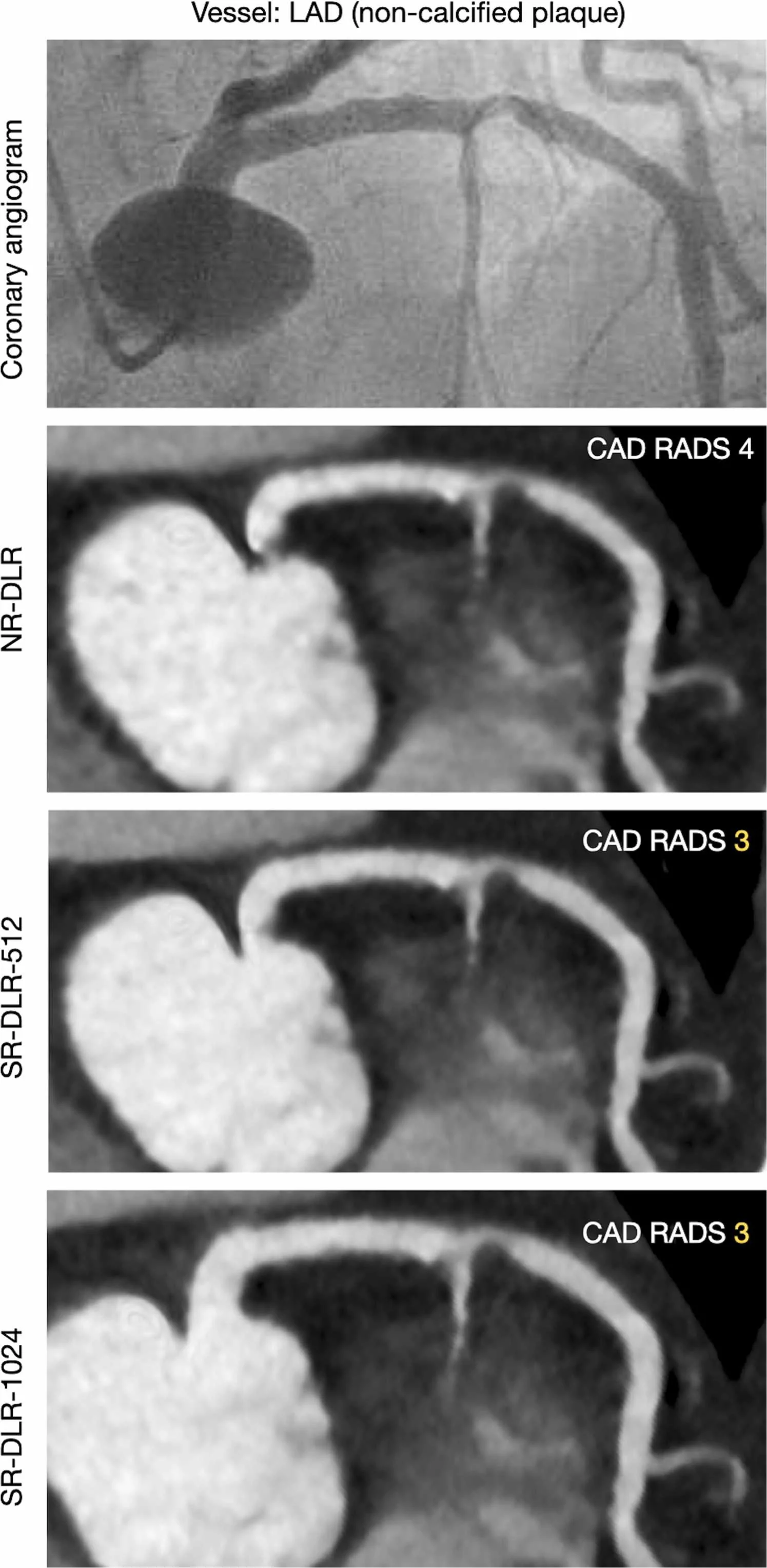
NR-DLR versus SR-DLR reconstructions in 512 and 1024 matrix and comparison with invasive coronary angiogram. A downgrading of CAD-RADS classification (from 4 to 3) was established between NR-DLR and SR-DLR (both 512 and 1024), which was comparable to the findings in the invasive coronary angiogram (50% stenosis). This is the only lesion of the cohort for which an invasive angiogram performed within 3 months was available; it is provided as an illustration and was not used as a reference standard.

**Fig. 4:**
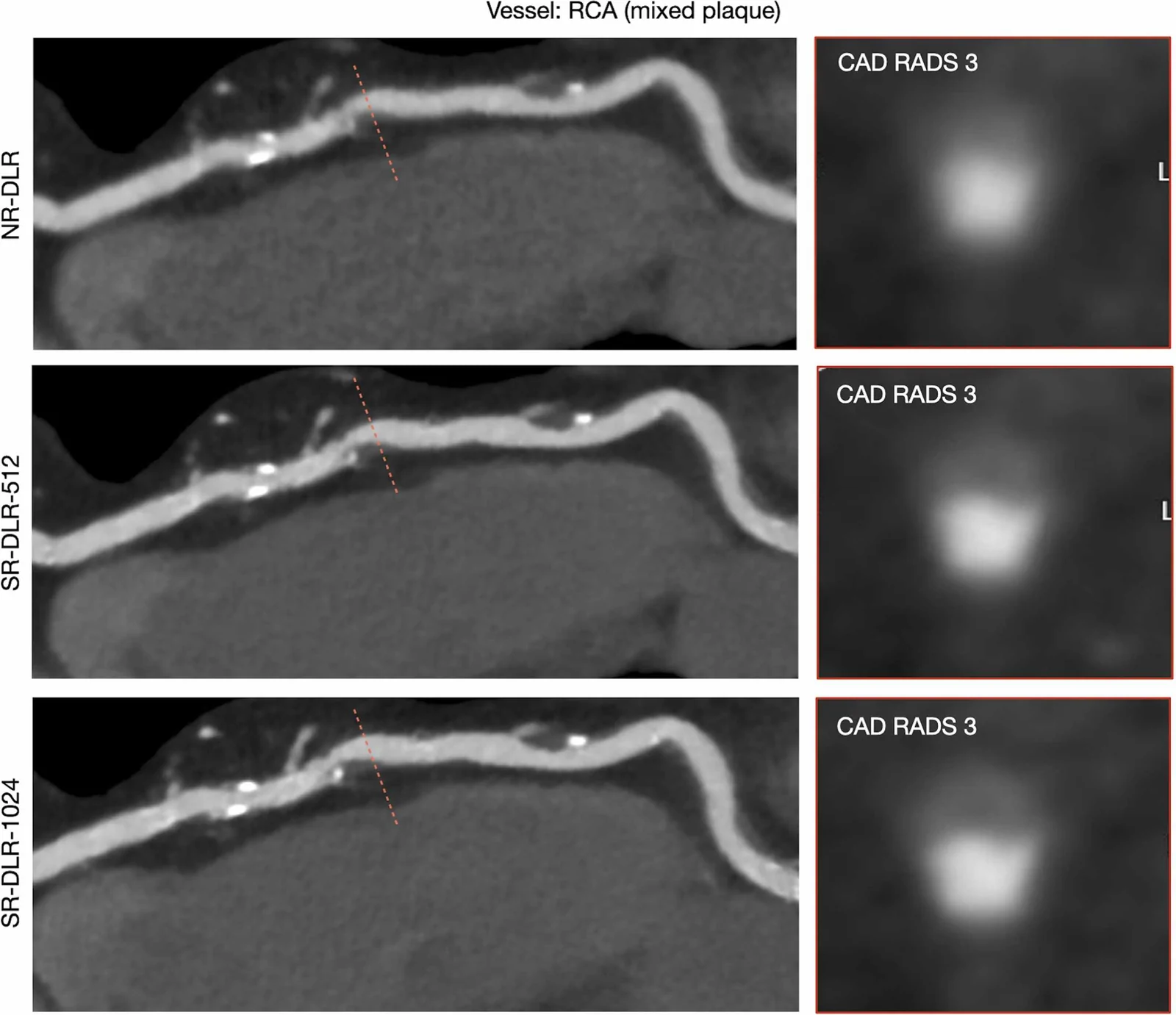
NR-DLR versus SR-DLR reconstructions in 512 and 1024 matrix. No downgrading of CAD-RADS classification was observed between NR-DLR and SR-DLR (512 and 1024). However, the boundaries of the non-calcified plaque components appear more clearly delineated with SR-DLR 512 and 1024.

**Fig. 5:**
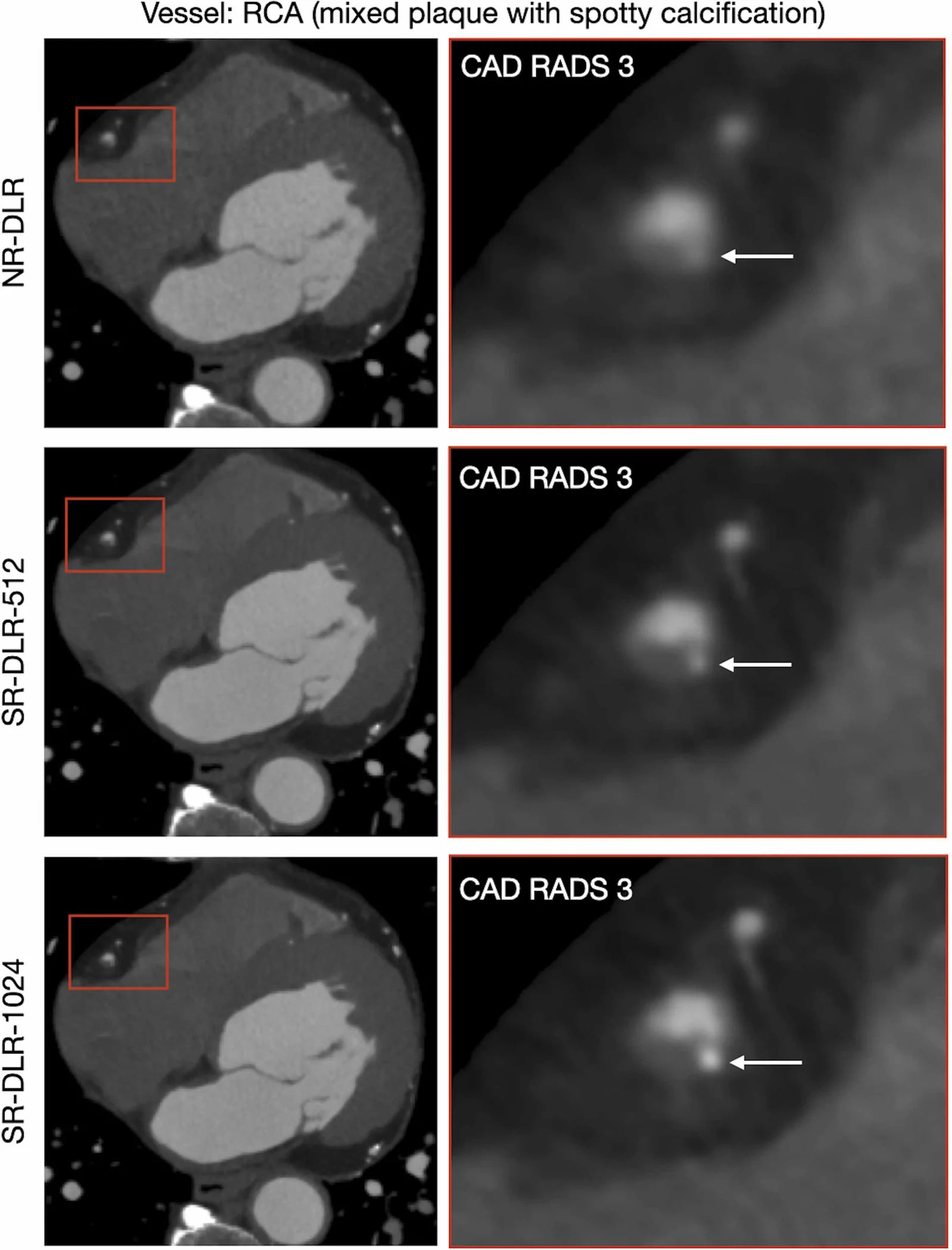
NR-DLR versus SR-DLR reconstructions in 512 and 1024 matrix. No downgrading of CAD-RADS classification was observed between NR-DLR and SR-DLR (512 and 1024). However, the spotty calcification (arrow) and the boundaries of the non-calcified plaque components are more clearly delineated with SR-DLR 512 and 1024.

**Fig. 6:**
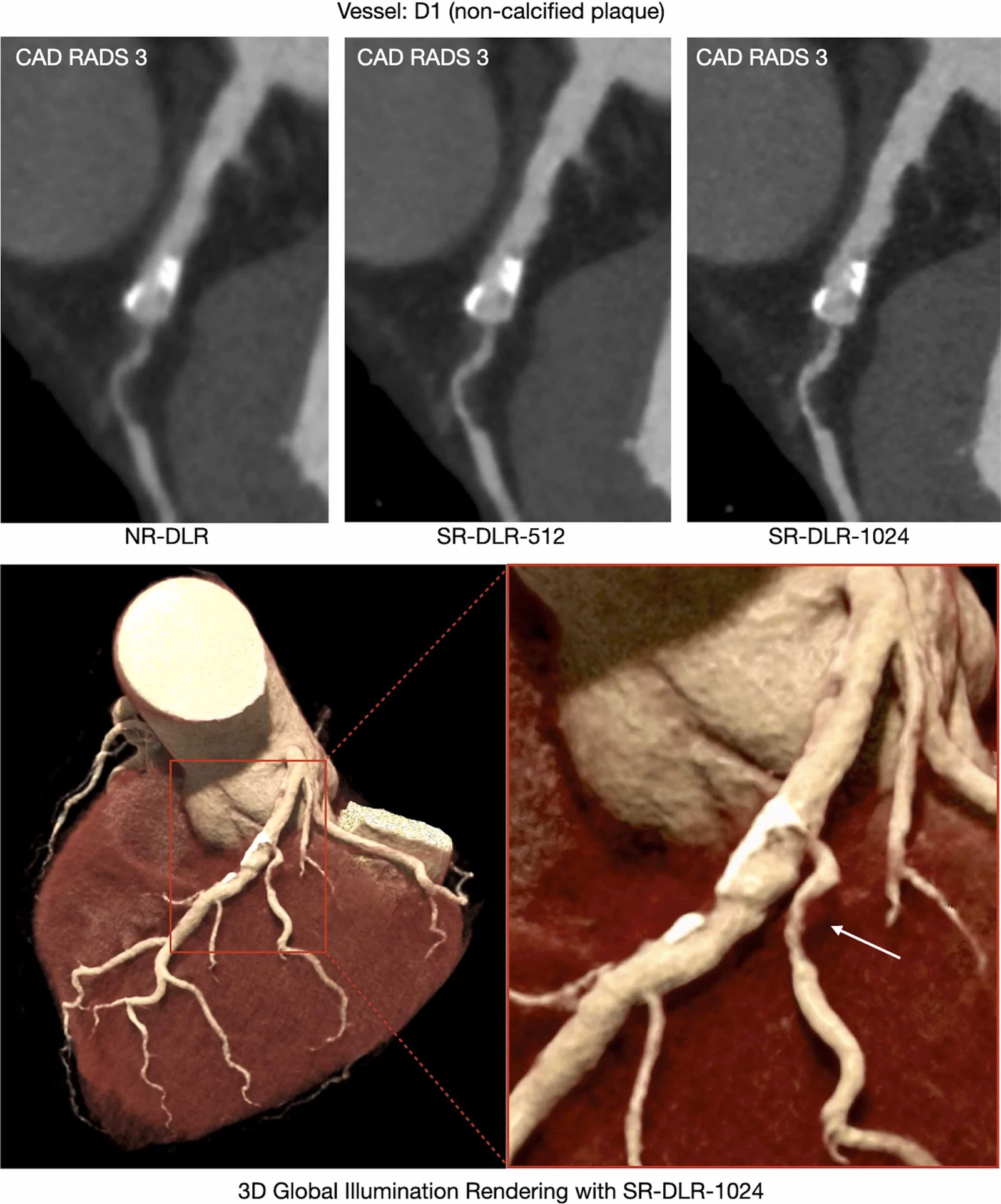
NR-DLR versus SR-DLR reconstructions in 512 and 1024 matrix. No downgrading of CAD-RADS classification was observed between NR-DLR and SR-DLR (512 and 1024). However, the residual lumen appears more clearly delineated with SR-DLR in both matrix sizes, and particularly well seen in 3D Global Illumination Rendering with SR-DLR-1024.

### 2.3 Expert reading

Five experienced readers with more than 10 years of expertise in CCTA, from 2 university hospitals assessed all included stenosis, independently, in a random order and as per local protocol, using a diagnostic workstation and a dedicated CCTA software analysis (Cardiac CTA coronaries, Vitrea, Canon Medical Informatics, Minnesota, U.S.A) with automatic curved MPR extraction. No automatic nor semi-automatic stenosis assessment tool was used. Visual stenosis assessment was intentionally performed according to routine clinical practice without predefined quantitative measurement thresholds, as the purpose of this study was to investigate whether different reconstruction strategies influence expert CAD-RADS classification under routine clinical reading conditions, CAD-RADS being defined as a visual, categorical classification in the 2.0 expert consensus document [1] and quantitative coronary CT measurements being currently reserved for research or core-laboratory settings [24]. Each reader evaluated all reconstructions independently; reconstructions from the same patient were separated in the randomization order to minimize recall bias.

To minimize recall bias, reconstructions originating from the same patient were distributed throughout the randomized reading list with a minimum separation of several unrelated cases. Readers were unaware that three reconstructions from the same patient were included in the dataset and were blinded to reconstruction type, clinical information and patient outcome.

For each dataset, the name of the vessel in which the potential CAD-RADS 3 lesion was initially identified was given to the readers. Every single reader had one task: grading the stenosis in the target vessel using the CAD-RADS classification. For all included coronary artery stenoses, 3 different CCTA reconstructions were available and were read independently and randomly by all 5 readers. All readers were blinded to patient clinical information and outcomes, as well as to the reconstruction type.

For every stenosis and each CT reconstruction (NR-DLR, SR-DLR-512 and SR-DLR-1024), the final consensus CAD-RADS grading was the one with the majority of readings (i.e. at least 3 out of 5 readers). When not attained, an additional expert reader was asked to independently grade the examination to achieve consensus; consensus was defined as a majority rule (>=3/5 readers).

### 2.4 Statistics

Descriptive statistics are presented as mean and standard deviations with ranges, and as medians with interquartile ranges when distributions were skewed, for continuous variables, and as counts with percentages and Wilson 95% confidence intervals (CI) for categorical variables. Inter-reader agreement was assessed using Gwet’s AC2 coefficient with quadratic weights between all five readers within each reconstruction group. According to commonly accepted interpretation thresholds for AC2, values above 0.80 were considered indicative of excellent agreement.

The rate of downgrading to a CAD-RADS 2 lesion was compared with an exact McNemar test:

- for each reader and for the consensus reading,

- with subgroup analysis based on the type of stenosis (calcified vs non-calcified), and on initial image quality

- for SR-DLR 512 and SR-DLR 1024, using NR-DLR as the reference reconstruction.

The McNemar test was applied only to paired reclassification rates and does not imply diagnostic accuracy, given the absence of a reference standard. The overall distribution of the three CAD-RADS categories between two reconstructions was additionally compared with the Stuart-Maxwell test of marginal homogeneity. Missing data were not imputed and the corresponding denominators are reported. At the reader level, the paired comparison was restricted to NR-DLR versus SR-DLR-1024, the primary comparison of interest, and reader-level proportions obtained with SR-DLR-512 are reported descriptively. Subgroup analyses (plaque composition, initial image quality) were exploratory and were not corrected for multiplicity; the corresponding p values are therefore descriptive. A two-sided p value < 0.05 was considered significant. Analyses were performed with R version 4.5.1 (R Foundation for Statistical Computing, Vienna, Austria).

### 2.5 Study design rationale

Because invasive coronary angiography was unavailable for this retrospective cohort, the objective of the present study was not to determine diagnostic accuracy but to evaluate whether SR-DLR modified visual CAD-RADS classification relative to standard reconstruction. The study should therefore be interpreted as an observer-performance investigation rather than a diagnostic accuracy study. This design is not a substitute for a diagnostic accuracy study but addresses a distinct and complementary question: whether a purely technical parameter, invisible to the reporting radiologist, is able to modify the CAD-RADS category and, consequently, the recommended patient pathway. Multi-reader observer-performance studies are the accepted methodology to quantify this specific effect.

## 3 Results

### 3.1 Study population

Thirty-nine CCTA examinations in 39 patients, totalizing 46 potential CAD-RADS 3 stenosis were ultimately included. This resulted in 138 (3 ×46) different reconstructed CCTA datasets (NR-DLR, SR-DLR 512 and SR-DLR 1024), that were analyzed by all 5 readers. Because the unit of analysis was the lesion, population characteristics are reported at the lesion level (n = 46). Sex ratio was 65.2% (30/46) men, mean age was 65.5 + /- 9.45 years old [45–90] (median 65.5, interquartile range 61.3–71.0) and mean weight was 78.1 + /- 20.9 kg [41−115] (median 80.6, interquartile range 68.0–95.0; available for 25/46 lesions, body height being available for 14/46 only, which precluded the computation of body mass index).

### 3.2 Coronary CT Angiography (CCTA)

Image quality of included CCTA was rated "good" in 75.6% (34/45), "moderate" in 15.6% (7/45) and "poor" in 8.9% (4/45); the initial quality rating was unavailable for one examination. Mean radiation dose (DLP) for the CCTA only (excluding the calcium score) was 188.4 + /- 171.7 mGy.cm [33−731] (median 148.5, interquartile range 67.0–280.5). Calcium score was positive in all lesions with available data, with a mean Agatston score of 680.7 + /- 638 [12–2363] (median 461.0, interquartile range 198.5–984.3; available for 44/46 lesions), corresponding to a median MESA percentile of 91.0 (interquartile range 82.5–96.5; available for 35/46 lesions).

Lesions were located in the LM in 4% (2/46), in the LAD in 63% (29/46) (Fig. 2, Fig. 3), in the Cx in 9% (4/46), in the RCA in 22% (10/46) (Fig. 4, Fig. 5), and in the D1 in 2% (1/46) (Fig. 6). All types of stenosis were represented: 19 calcified, 16 mixed, 10 non-calcified and 1 in-stent.

### 3.3 Expert reading

For all 138 datasets, consensus CAD-RADS ranking ranged from 2 to 4; at the individual reader level, a single reading was graded CAD-RADS 5. A consensus was always achievable between all 5 readers and for all 138 datasets (3 ×46 cases), without the need for an additional reader.

#### 3.3.1 Inter-reader agreement

Inter-reader reproducibility remained excellent irrespective of the reconstruction method. Gwet’s AC2 coefficients were 0.89 (95% CI: 0.86–0.92) for NR-DLR, 0.87 (95% CI: 0.83–0.90) for SR-DLR-512 and 0.84 (95% CI: 0.81–0.88) for SR-DLR-1024. Although a slight numerical decrease in agreement was observed with increasing reconstruction resolution, all reconstruction methods remained within the range generally considered to represent excellent agreement (Table 3), and the confidence intervals of the three coefficients overlapped.

### 3.4 CAD-RADS reclassification

#### 3.4.1 Overall consensus analysis

Consensus CAD-RADS distributions for the three reconstruction methods are summarized in Table 2. Compared with conventional NR-DLR reconstruction, SR-DLR reconstructed with a 1024 matrix resulted in a greater proportion of lesions being classified as CAD-RADS 2. Specifically, consensus CAD-RADS 2 classifications increased from 7% (3/46) using NR-DLR to 22% (10/46) using SR-DLR-1024, whereas the proportion of CAD-RADS 3 lesions decreased from 78% (36/46) to 65% (30/46). The proportion of CAD-RADS 4 lesions remained essentially unchanged (15% versus 13%). Overall, lesion classification remained unchanged in 34/46 examinations (74%), increased in 2/46 examinations (4%), and decreased in 10/46 examinations (22%) when comparing SR-DLR-1024 with NR-DLR. The increase in the proportion of lesions classified CAD-RADS 2 was significant (exact McNemar test, p = 0.016), as was the overall shift of the category distribution (Stuart-Maxwell test, p = 0.027). The individual flow of reclassifications is displayed as a Sankey diagram in Fig. 7: seven lesions moved from CAD-RADS 3 to CAD-RADS 2, three from CAD-RADS 4 to CAD-RADS 3, and two from CAD-RADS 3 to CAD-RADS 4. Among downgraded lesions, non-calcified plaques were overrepresented, whereas the few lesions upgraded from CAD-RADS 3 to CAD-RADS 4 were exclusively calcified. With SR-DLR-512, reclassification was less frequent and not significant: six lesions were downgraded and three upgraded, the proportion of CAD-RADS 2 lesions increasing from 6.5% (3/46) to 10.9% (5/46) (exact McNemar p = 0.625; Stuart-Maxwell p = 0.549).

**Tablee 2:** Consensus CAD-RADS grading according to the reconstruction method.

	**NR-DLR (n = 46)**	**SR-DLR-512 (n = 46)**	**SR-DLR-1024 (n = 46)**
CAD-RADS 2	3 (7%) [2.2–17.5]	5 (11%) [4.7–23.0]	10 (22%) [12.3–35.6]
CAD-RADS 3	36 (78%) [64.4–87.7]	35 (76%) [62.1–86.1]	30 (65%) [50.8–77.3]
CAD-RADS 4	7 (15%) [7.6–28.2]	6 (13%) [6.1–25.7]	6 (13%) [6.1–25.7]

Data are n (%) [Wilson 95% confidence interval]. NR-DLR versus SR-DLR-1024: exact McNemar test on the proportion of CAD-RADS 2 lesions, p = 0.016; Stuart-Maxwell test of marginal homogeneity on the three categories, p = 0.027. NR-DLR versus SR-DLR-512: p = 0.625 and p = 0.549, respectively. CAD-RADS = Coronary Artery Disease - Reporting and Data System; NR-DLR = normal-resolution deep learning reconstruction; SR-DLR = super-resolution deep learning reconstruction.

**Table 3:** Inter-reader agreement.

**CT reconstruction**	**Gwet’s AC2 coefficient [95% CI]**
NR-DLR	0.89 [0.86; 0.92]
SR-DLR-512	0.87 [0.83; 0.90]
SR-DLR-1024	0.84 [0.81; 0.88]

Agreement computed between the five readers, with quadratic weights. AC2 = Gwet’s second-order agreement coefficient; CI = confidence interval; CT = computed tomography; NR-DLR = normal-resolution deep learning reconstruction; SR-DLR = super-resolution deep learning reconstruction.

**Fig. 7:**
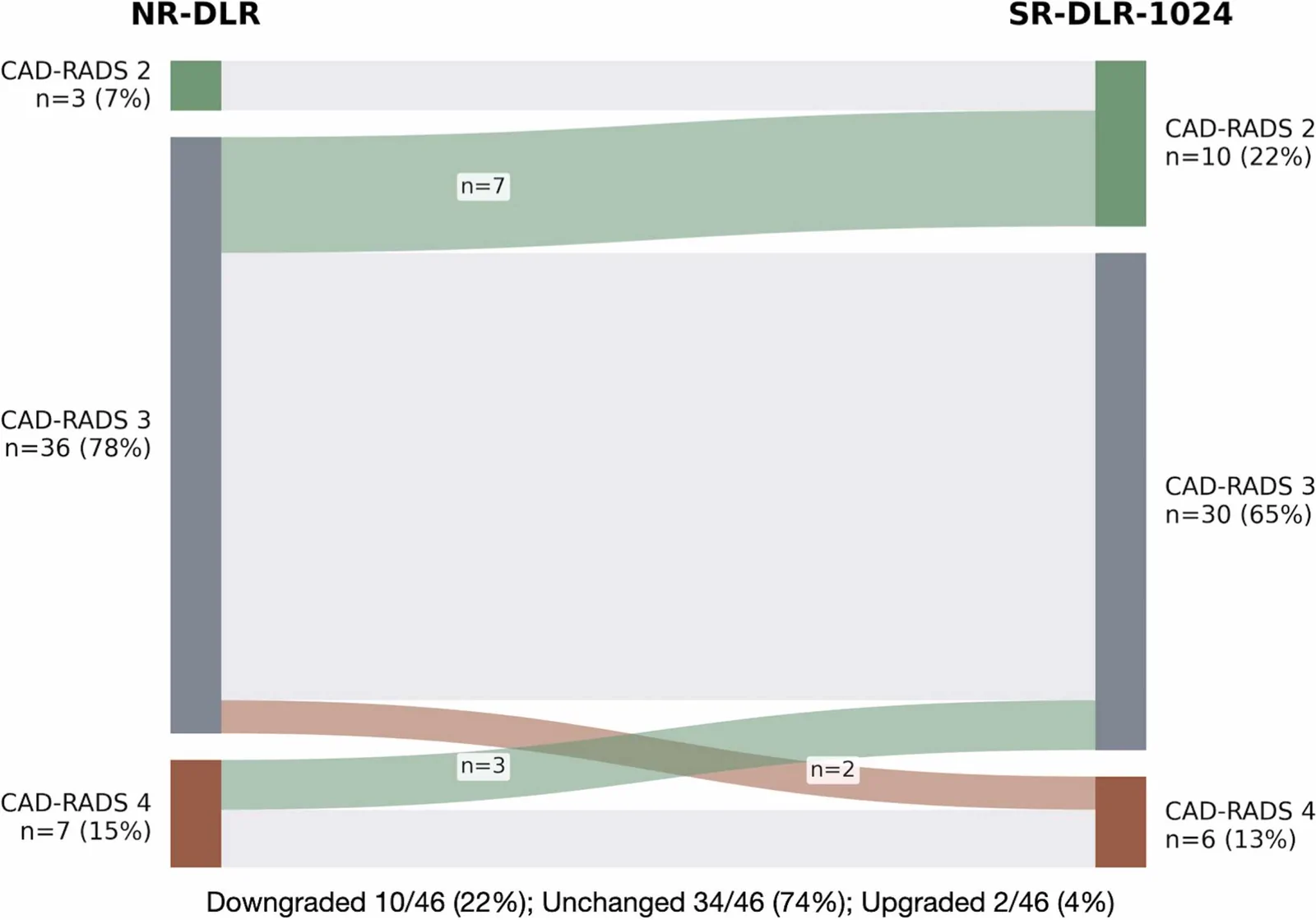
Sankey diagram of consensus CAD-RADS reclassification between NR-DLR (reference) and SR-DLR-1024 for the 46 analysed lesions. Green ribbons indicate downgrading, red ribbons upgrading and grey ribbons unchanged categories. NR-DLR = normal-resolution deep learning reconstruction; SR-DLR = super-resolution deep learning reconstruction.

#### 3.4.2 Individual reader analysis

The proportion of lesions classified CAD-RADS 2 by each reader and for each reconstruction is summarized in Table 4. Two of the five readers demonstrated a statistically significant increase in the proportion of CAD-RADS 2 lesions between NR-DLR and SR-DLR-1024 (Reader 1, from 6.5% to 26.1%, p = 0.004; Reader 3, from 0% to 19.6%, p = 0.004), whereas Reader 2 showed the same trend without reaching significance (4.3–13.0%, p = 0.219). Reader 4 was unchanged (28.3% versus 28.3%, p = 1.00) and Reader 5 showed a non-significant decrease (39.1–30.4%, p = 0.388).

**Table 4:** Proportion of lesions classified CAD-RADS 2 by each reader, according to the reconstruction method (n = 46 lesions).

**Reader**	**NR-DLR**	**SR-DLR-512**	**SR-DLR-1024**	**p value**
R1	3 (6.52)	9 (19.57)	12 (26.09)	0.004
R2	2 (4.35)	4 (8.70)	6 (13.04)	0.219
R3	0 (0.00)	4 (8.70)	9 (19.57)	0.004
R4	13 (28.26)	10 (21.74)	13 (28.26)	1.00
R5	18 (39.13)	17 (36.96)	14 (30.43)	0.388

Data are n (%) of the 46 analysed lesions. The p value corresponds to the exact McNemar test comparing the paired proportions of CAD-RADS 2 lesions obtained with NR-DLR and with SR-DLR-1024, the primary comparison of interest; proportions obtained with SR-DLR-512 are reported descriptively. CAD-RADS = Coronary Artery Disease - Reporting and Data System; NR-DLR = normal-resolution deep learning reconstruction; R = reader; SR-DLR = super-resolution deep learning reconstruction.

Substantial inter-reader variability was observed, and it was already present on the reference reconstruction: with NR-DLR, the proportion of lesions graded CAD-RADS 2 ranged from 0% (Reader 3) to 39.1% (Reader 5). The two readers in whom SR-DLR-1024 significantly increased downgrading were precisely those with the lowest baseline rates, whereas the two readers who already assigned CAD-RADS 2 to more than a quarter of the lesions on NR-DLR showed no further increase. At the marginal level, this pattern is compatible with a ceiling effect, although stable marginal proportions concealed substantial bidirectional changes at the lesion level (Reader 4: 8 lesions newly graded CAD-RADS 2 and 8 no longer graded CAD-RADS 2 with SR-DLR-1024; Reader 5: 4 and 8, respectively). Overall, these observations indicate that the impact of the reconstruction on the CAD-RADS category depends on the individual grading threshold of the reader. With SR-DLR-1024, the proportion of CAD-RADS 2 lesions ranged from 13.0% to 30.4% across readers, i.e. a narrower dispersion than with NR-DLR (0–39.1%).

#### 3.4.3 Plaque composition

Exploratory subgroup analysis suggested that lesion reclassification occurred predominantly among non-calcified plaques (Table 5). In the 27 lesions containing a non-calcified component (10 purely non-calcified, 16 mixed and one in-stent restenosis), the proportion of CAD-RADS 2 lesions increased from 7.4% (2/27) with NR-DLR to 25.9% (7/27) with SR-DLR-1024, a difference that approached but did not reach significance (exact McNemar p = 0.063). In the 19 calcified lesions, the corresponding proportions were 5.3% (1/19) and 15.8% (3/19) (p = 0.50). Calcified plaques were comparatively stable across reconstruction methods, although qualitative reduction of blooming artefacts was visually observed in several cases reconstructed using SR-DLR-1024. Because subgroup sample sizes were limited, particularly for non-calcified plaques and the single in-stent restenosis, these observations should be considered exploratory and the absence of statistical significance within subgroups must not be interpreted as an absence of effect.

**Table 5:** Exploratory subgroup analysis: proportion of lesions classified CAD-RADS 2 by consensus, according to plaque composition and initial image quality.

**Subgroup**	**n**	**NR-DLR, n (%)**	**SR-DLR-1024, n (%)**	**p value**
Lesions with a non-calcified component	27	2 (7.4)	7 (25.9)	0.063
Calcified lesions	19	1 (5.3)	3 (15.8)	0.50
Image quality rated good	34	2 (5.9)	8 (23.5)	0.031
Image quality rated moderate or poor	11	1 (9.1)	2 (18.2)	1.00

Data are n (%) of the lesions of each subgroup. The subgroup of lesions with a non-calcified component includes purely non-calcified plaques (n = 10), mixed plaques (n = 16) and the single in-stent restenosis (n = 1). Image quality was rated at the initial clinical reading and was unavailable for one examination. p values correspond to exact McNemar tests and are not corrected for multiplicity. CAD-RADS = Coronary Artery Disease - Reporting and Data System; NR-DLR = normal-resolution deep learning reconstruction; SR-DLR = super-resolution deep learning reconstruction.

#### 3.4.4 Initial image quality

An additional exploratory subgroup analysis was performed according to the image quality rated at the initial clinical reading (Table 5). Among the 34 lesions from examinations rated "good", the proportion of lesions classified CAD-RADS 2 increased significantly from 5.9% (2/34) with NR-DLR to 23.5% (8/34) with SR-DLR-1024 (exact McNemar p = 0.031). Among the 11 lesions from examinations rated "moderate" or "poor", the corresponding proportions were 9.1% (1/11) and 18.2% (2/11) (p = 1.00). Although the size of the second subgroup precludes any firm conclusion, this pattern suggests that the influence of the reconstruction matrix on CAD-RADS classification is mainly expressed when image quality is not already degraded by noise or motion.

### 3.5 Qualitative image assessment

Across multiple cases, SR-DLR reconstructed with a 1024 matrix appeared to improve visualization of the residual coronary lumen and the plaque-lumen interface. Blooming artefacts appeared visually less pronounced in selected calcified lesions, whereas plaque margins appeared more sharply defined in several non-calcified lesions. Representative examples are illustrated in Fig. 2, Fig. 3, Fig. 4, Fig. 5, Fig. 6. No quantitative measurements of blooming width, edge sharpness or plaque morphology were performed; therefore, these observations remain qualitative.

## 4 Discussion

The present study demonstrates that image reconstruction strategy influences visual CAD-RADS classification in intermediate coronary stenoses. In a cohort intentionally enriched with lesions initially classified as CAD-RADS 3 on conventional reconstruction, SR-DLR reconstructed with a 1024 matrix resulted in more frequent downgrading to CAD-RADS 2 than conventional deep learning reconstruction while preserving excellent inter-reader agreement. These findings suggest that image reconstruction should no longer be regarded solely as a technical post-processing step but also as a factor capable of influencing visual interpretation and CAD-RADS classification in clinically challenging lesions.

The objective of the present work was deliberately different from that of a diagnostic accuracy study. Rather than determining whether SR-DLR provides a more accurate estimation of coronary stenosis severity, we sought to establish whether advanced reconstruction modifies reader interpretation in lesions located at one of the most clinically relevant diagnostic thresholds. Consequently, the observed reclassification should not be interpreted as evidence of improved diagnostic accuracy. Because invasive coronary angiography, quantitative coronary angiography and physiological assessment were unavailable, the present study cannot determine whether lesion downgrading reflects more accurate lumen assessment or altered visual perception induced by improved image characteristics. Nevertheless, demonstrating that reconstruction-induced reclassification occurs at the consensus level and in several experienced readers, albeit with marked inter-reader heterogeneity, represents an important observation and provides the basis for future diagnostic validation.

Three arguments nonetheless make a purely artefactual explanation of our findings unlikely. First, reclassification was bidirectional: two lesions were upgraded from CAD-RADS 3 to CAD-RADS 4 with SR-DLR-1024, which is incompatible with a systematic bias of the algorithm towards apparently smaller stenoses. Second, the physical behaviour of the algorithm is documented in phantom studies with a known ground truth, in which SR-DLR improves the task-transfer function and edge definition [9], [13], [14], [18], [19]; reclassification is therefore consistent with an expected reduction of partial-volume and blooming effects. Third, the same phenomenon has been described with hardware-based increases in spatial resolution (UHR-CT and photon-counting CT), where downgrading was validated against invasive angiography [5], [6], [7], [8], [25], [26]. This convergence does not prove accuracy, and the residual possibility that a generative reconstruction produces plausible but inaccurate lumen contours must be acknowledged; it does, however, support a physically plausible mechanism rather than a pure algorithmic artefact.

Our findings should be interpreted alongside the rapidly growing literature on ultra-high-resolution coronary imaging. Most notably, Zou et al. recently reported the first prospective multicenter evaluation of SR-DLR using invasive coronary angiography as the reference standard, demonstrating CAD-RADS reclassification in approximately one fifth of patients together with improved diagnostic performance [23]. Although our study differs substantially in design, notably by not including invasive validation, it also differs technically. Whereas Zou et al. compared SR-DLR reconstructed with a 512 matrix against hybrid iterative reconstruction, our study compared SR-DLR reconstructed with both 512 and 1024 matrices against normal-resolution deep learning reconstruction (NR-DLR), representing a more demanding comparison against an already advanced reconstruction algorithm. Furthermore, our study specifically evaluated the additional influence of 1024-matrix reconstruction on reader interpretation in lesions intentionally enriched for intermediate CAD-RADS 3 stenoses, rather than overall diagnostic accuracy. Despite these methodological differences, the same direction and a comparable order of magnitude of CAD-RADS reclassification strengthen the reproducibility of this phenomenon across independent patient populations. Importantly, our results should therefore be viewed as complementary to those of Zou et al. Rather than confirming improved diagnostic accuracy, they independently demonstrate that reconstruction-induced changes in CAD-RADS categorization are reproducible. Together, these findings support the hypothesis that reconstruction strategy represents an independent determinant of coronary stenosis interpretation, while highlighting the need for future studies integrating both diagnostic accuracy and observer variability.

Interestingly, similar observations have now been reported using three independent approaches to improving effective spatial resolution: dedicated ultra-high-resolution CT, photon-counting CT and software-based super-resolution reconstruction [4], [5], [6], [7], [8], [23], [25]. Halfmann et al. and Vecsey-Nagy et al. both reported frequent CAD-RADS downgrading with ultra-high-resolution imaging [8], [25]. Taken together, these studies suggest that reconstruction-induced reclassification is unlikely to represent a manufacturer-specific phenomenon. Instead, they support a broader relationship between effective spatial resolution and visual stenosis assessment, regardless of whether higher resolution is achieved through detector technology or reconstruction algorithms.

The mechanisms underlying these observations are likely multifactorial. Previous phantom and clinical studies have consistently shown that SR-DLR improves spatial resolution, edge sharpness and noise characteristics, thereby enhancing visualization of the residual coronary lumen and plaque-lumen interface [9], [10], [11], [12], [13], [14], [15], [16], [17], [18], [19], [20], [21], [22]. Such improvements may influence visual estimation of stenosis severity, particularly when lesions lie close to the threshold separating CAD-RADS 2 from CAD-RADS 3. Interestingly, reclassification was numerically more frequent in lesions with a non-calcified component, in which the proportion of CAD-RADS 2 lesions increased from 7.4% to 25.9% (p = 0.063), compared with 5.3–15.8% in calcified lesions (p = 0.50), suggesting that improved plaque-lumen interface delineation may contribute in addition to any reduction in calcium blooming. The increase was also significant only in examinations of good initial quality (p = 0.031), which, although subgroup sizes preclude firm conclusions, is compatible with a resolution-driven mechanism: when noise or motion dominate, a finer sampling grid cannot restore the plaque-lumen interface. Objective image-quality indices were deliberately not measured on patient data: region-of-interest noise depends on the pixel size, so that signal-to-noise and contrast-to-noise ratios are neither directly comparable between the 512 and 1024 matrices nor surrogates of spatial resolution for a non-linear, resolution-oriented reconstruction, whereas the relevant task-based metrics (task-transfer function, noise-power spectrum, edge profiles) require phantom conditions and have already been reported for this algorithm, including with the 1024 matrix [9], [13], [14], [18], [19]. Because blooming width, lumen dimensions and plaque morphology were not quantitatively assessed, these explanations remain hypothetical and should be interpreted as plausible mechanisms rather than direct conclusions of the present study.

The choice of a purely visual grading also deserves comment. CAD-RADS 2.0 is explicitly defined as a visual and categorical classification [1], and the SCCT expert consensus on quantitative CCTA assessment restricts calliper-based or semi-automated measurements to research and core-laboratory settings, mainly because of their limited reproducibility on borderline lesions [24]. In addition, quantitative measurements performed on reconstructions with different pixel sizes (0.5 mm versus 0.25 mm) would themselves be influenced by the matrix under evaluation, thereby confounding the comparison. More fundamentally, lumen contours measured on a given reconstruction are themselves derived from that reconstruction, so that quantitative measurements could not distinguish a true difference in stenosis severity from an algorithm-dependent change in lumen depiction; only an external reference standard can make this distinction. Since our objective was precisely to capture what a reporting radiologist decides in routine conditions, visual grading was the appropriate and internally consistent choice. We nevertheless acknowledge that the absence of quantitative diameter measurements prevents any estimation of the magnitude, in millimetres or in percent diameter stenosis, of the observed reclassifications.

High-risk plaque features, which carry prognostic value independently of stenosis severity [2], [3], were not recorded during the blinded reading sessions, which were deliberately designed as a single-task experiment, and could not be reassessed post hoc without breaking the original blinding conditions, since the readers are no longer naive to the study hypothesis. Individual high-risk features were nevertheless qualitatively more conspicuous on SR-DLR-1024 in several cases, in particular the napkin-ring sign (Fig. 1) and spotty calcification (Fig. 5). Because a reconstruction-induced change in the detection of these features would affect risk stratification, a dedicated prospective evaluation of the impact of super-resolution reconstruction on high-risk plaque characterisation, ideally against intravascular imaging, is warranted.

The per-reader analysis also deserves attention, because it nuances the apparent stability of the consensus. On the reference reconstruction itself, the proportion of lesions graded CAD-RADS 2 ranged from 0% to 39% depending on the reader, and the two readers in whom SR-DLR-1024 significantly increased downgrading were those with the most conservative baseline threshold. Two observations follow. First, the effect of the reconstruction is not uniform across observers but interacts with each reader’s individual decision threshold, which is an argument for reporting observer-level results rather than consensus alone. Second, the high Gwet’s AC2 values must be interpreted in the light of these marginal distributions: agreement coefficients quantify concordance on individual lesions, and the concentration of ratings within the CAD-RADS 3 category, together with the quadratic weighting, contributes to high coefficients despite substantial differences between reader marginals. Gwet’s AC2 was chosen precisely because it is robust to this high-agreement paradox, but the coexistence of excellent AC2 values and heterogeneous individual thresholds is in itself an informative finding.

One of the principal strengths of our study is its deliberate focus on intermediate coronary stenoses. Previous investigations have generally evaluated SR-DLR across the entire spectrum of coronary artery disease, whereas we specifically targeted the subgroup in which relatively small differences in visual interpretation are most likely to influence CAD-RADS classification. To our knowledge, this is among the first studies specifically investigating reconstruction-induced CAD-RADS reclassification in a cohort intentionally enriched with intermediate coronary stenoses. Furthermore, we directly compared two reconstruction matrices using the same reconstruction algorithm and evaluated observer performance across five experienced readers from two tertiary centers. These methodological features provide original information regarding the interaction between reconstruction parameters and reader behavior beyond conventional image quality assessment.

Our findings also have methodological implications. CAD-RADS was developed to standardize reporting and guide patient management [1]. Consequently, any technical factor capable of modifying lesion categorization deserves consideration. Although our data do not support changes in clinical decision-making in the absence of invasive validation, they indicate that reconstruction strategy should be acknowledged as a potential source of variability when interpreting borderline coronary stenoses or when comparing CAD-RADS classifications across reconstruction protocols or longitudinal imaging studies. Future studies should therefore focus less on demonstrating whether advanced reconstruction modifies CAD-RADS classification, which is now reported across several independent studies, and more on determining whether these reconstruction-induced changes improve diagnostic accuracy and clinical outcomes.

Overall, the present findings should be considered hypothesis-generating. They support further prospective studies incorporating invasive reference standards, quantitative plaque analysis and clinical outcomes to determine whether reconstruction-induced CAD-RADS reclassification translates into improved patient management.

Several limitations should be acknowledged. First, the absence of invasive reference standards precludes assessment of diagnostic accuracy; our results describe reader behaviour and must not be read as evidence that SR-DLR-1024 is closer to the truth than NR-DLR. Second, the study population was intentionally restricted to lesions initially classified as CAD-RADS 3, limiting extrapolation to unselected CCTA populations but allowing focused investigation of the subgroup in which reconstruction effects are expected to be greatest. Third, despite randomization, complete elimination of recognition bias cannot be guaranteed because readers evaluated three reconstructions from each examination. Fourth, neither high-risk plaque features nor objective image-quality metrics were assessed, so that the mechanistic interpretation of our findings relies on published phantom evidence. Fifth, subgroup analyses were exploratory, were not corrected for multiplicity and were underpowered, so that the absence of significance within a subgroup cannot be interpreted as an absence of effect. Sixth, anthropometric data were incompletely available in this retrospective cohort (body weight in 25/46 and body height in 14/46 lesions), which precluded any analysis according to body mass index. Finally, the relatively small sample size, single-vendor design and absence of quantitative analysis of blooming or plaque morphology limit mechanistic interpretation and generalizability. Moreover, because only one commercially available SR-DLR algorithm was evaluated, the present findings cannot automatically be generalized to other deep learning reconstruction techniques.

## 5 Conclusion

In conclusion, SR-DLR reconstructed with a 1024 matrix modified CAD-RADS classification in a clinically relevant proportion of intermediate coronary stenoses while maintaining excellent inter-reader agreement. Rather than demonstrating improved diagnostic accuracy, the present study provides further evidence that image reconstruction itself influences visual CAD-RADS categorization. Together with previous studies using ultra-high-resolution CT, photon-counting CT and SR-DLR [4], [5], [6], [7], [8], [23], [25], [26], these findings support the concept that reconstruction strategy has become an important component of coronary CTA interpretation and should be considered when evaluating borderline coronary stenoses. Whether these reconstruction-induced changes ultimately translate into improved diagnostic accuracy, more appropriate clinical decision-making and better patient outcomes remains to be established in prospective studies incorporating invasive reference standards and clinical outcome measures.

## Ethics declaration

The authors have NOT obtained informed consent from participants or their legal representatives. Institutional Review Board (Strasbourg University Hospital) approval (CE-2023–89) was obtained for the present study. Given its retrospective nature, signed patient consent was waived.

This study was performed in compliance with relevant laws, regulatory frameworks and guidelines where the research took place. This study was approved by the Strasbourg University Hospital. (Approval No. CE-2023–89)

## Study in humans, informed consent and patient details

Institutional Review Board approval was obtained for the present study. Given its retrospective nature, signed patient consent was waived.

## Declaration of Generative AI and AI-assisted technologies in the writing process

The authors used ChatGPT-4 (OpenAI) to improve the clarity and fluency of the English language during the writing of this manuscript. After using this tool, the authors reviewed and edited the content as needed and take full responsibility for the content of the publication.

## Fundings

Mickael Ohana and Kazuo Awai received research grant from 10.13039/100015650Canon Medical Systems Corporation.

## Declaration of Competing Interest

Mickael Ohana (MO) and Kazuo Awai (KA) received research grant from Canon Medical Systems Corporation. Akira Taniguchi (AT) and Karim Haioun (KH) is an employee of Canon Medical Systems Corporation. This affiliation may represent a potential conflict of interest. The other authors (JPD, FM, AL, WF, SEG, CR) have no conflicts of interest to declare.

## References

[1] Cury R.C., Leipsic J., Abbara S. (2022). CAD-RADS 2.0–2022 coronary artery disease–reporting and data system: an expert consensus document of the SCCT, ACC, ACR, and NASCI. JACC Cardiovasc. Imaging.

[2] Conte E., Andreini D., Magnoni M. (2021). Association of high-risk coronary atherosclerosis at CCTA with clinical and circulating biomarkers: insight from CAPIRE study. J. Cardiovasc. Comput. Tomogr..

[3] Lee S.E., Chang H.J., Rizvi A. (2016). Rationale and design of the Progression of AtheRosclerotic PlAque DetermIned by Computed TomoGraphic Angiography IMaging (PARADIGM) registry: a comprehensive exploration of plaque progression and its impact on clinical outcomes from a multicenter serial coronary computed tomographic angiography study. Am. Heart J..

[4] Oostveen L.J., Boedeker K.L., Brink M. (2020). Physical evaluation of an ultra-high-resolution CT scanner. Eur. Radiol..

[5] Takagi H., Tanaka R., Nagata K. (2018). Diagnostic performance of coronary CT angiography with ultra-high-resolution CT: comparison with invasive coronary angiography. Eur. J. Radiol..

[6] Fukui R., Harashima S., Samejima W. (2025). Acquisition and reconstruction techniques for coronary CT angiography: current status and trends over the past decade. Radiographics.

[7] Koons E.K., Rajiah P.S., Thorne J.E. (2024). Coronary artery stenosis quantification in patients with dense calcifications using ultra-high-resolution photon-counting-detector CT. J. Cardiovasc. Comput. Tomogr..

[8] Halfmann M.C., Bockius S., Emrich T. (2024). Ultrahigh-spatial-resolution photon-counting detector CT angiography of coronary artery disease for stenosis assessment. Radiology.

[9] Higaki T., Tatsugami F., Ohana M. (2024). Super resolution deep learning reconstruction for coronary CT angiography: a structured phantom study. Eur. J. Radiol. Open..

[10] Latina J., Shabani M., Kapoor K. (2021). Ultra-high-resolution coronary CT angiography for assessment of patients with severe coronary artery calcification: initial experience. Radiol. Cardiothorac. Imaging.

[11] Gillet R., Boubaker F., Hossu G. (2023). Computed tomography bone imaging: pushing the boundaries in clinical practice. Semin. Musculoskelet. Radiol..

[12] Greffier J., Viry A., Robert A. (2025). Photon-counting CT systems: a technical review of current clinical possibilities. Diagn. Interv. Imaging.

[13] Greffier J., Dabli D., de Oliveira F. (2025). Contribution of a new super-resolution deep-learning image reconstruction algorithm coupled with an extended matrix to cardiac CT image quality. Diagn. Interv. Imaging.

[14] Greffier J., Pastor M., Si-Mohamed S. (2024). Comparison of two deep-learning image reconstruction algorithms on cardiac CT images: a phantom study. Diagn. Interv. Imaging.

[15] Takafuji M., Kitagawa K., Mizutani S. (2023). Super-resolution deep learning reconstruction for improved image quality of coronary CT angiography. Radiol. Cardiothorac. Imaging.

[16] Tatsugami F., Higaki T., Kawashita I. (2023). Improvement of spatial resolution on coronary CT angiography by using super-resolution deep learning reconstruction. Acad. Radiol..

[17] Nagayama Y., Emoto T., Kato Y. (2023). Improving image quality with super-resolution deep-learning-based reconstruction in coronary CT angiography. Eur. Radiol..

[18] Sawamura S., Kato S., Funama Y. (2024). Evaluation of four computed tomography reconstruction algorithms using a coronary artery phantom. Quant. Imaging Med. Surg..

[19] Sato H., Fujimoto S., Tomizawa N. (2023). Impact of a deep learning-based super-resolution image reconstruction technique on high-contrast computed tomography: a phantom study. Acad. Radiol..

[20] Orii M., Sone M., Osaki T. (2023). Super-resolution deep learning reconstruction at coronary computed tomography angiography to evaluate the coronary arteries and in-stent lumen: an initial experience. BMC Med. Imaging.

[21] Zou L.M., Xu C., Xu M. (2025). Ultra-low-dose coronary CT angiography via super-resolution deep learning reconstruction: impact on image quality, coronary plaque, and stenosis analysis. Eur. Radiol..

[22] Matsuyama T., Nagata H., Ozawa Y. (2025). High-resolution deep learning reconstruction for coronary CTA: compared efficacy of stenosis evaluation with other methods at in vitro and in vivo studies. Eur. Radiol..

[23] Zou L., Xu C., Liu X. (2026). Super-Resolution Deep Learning Reconstruction for Coronary CT Angiography: Coronary Stenosis Assessment and CAD-RADS Reclassification. Radiology.

[24] Nieman K., García-García H.M., Hideo-Kajita A. (2024). Standards for quantitative assessments by coronary computed tomography angiography (CCTA): an expert consensus document of the SCCT. J. Cardiovasc. Comput. Tomogr..

[25] Vecsey-Nagy M., Tremamunno G., Schoepf U.J. (2024). Intraindividual comparison of ultrahigh-spatial-resolution photon-counting detector CT and energy-integrating detector CT for coronary stenosis measurement. Circulation Cardiovascular Imaging.

[26] Vecsey-Nagy M., Varga-Szemes A., Schoepf U.J., Tremamunno G., Fink N., Zsarnoczay E., Emrich T. (2024). Ultra-high resolution coronary CT angiography on photon-counting detector CT: bi-centre study on the impact of quantum iterative reconstruction on image quality and accuracy of stenosis measurements. Eur. J. Radiol..

